# Predictors of progression from a first demyelinating event to clinically definite multiple sclerosis

**DOI:** 10.1093/braincomms/fcac181

**Published:** 2022-07-09

**Authors:** Caron Chapman, Robyn M Lucas, Anne-Louise Ponsonby, Bruce Taylor, Ausimmune Investigator Group, Caron Chapman, Alan Coulthard, Keith Dear, Terry Dwyer, Trevor Kilpatrick, Robyn Lucas, Tony McMichael, Michael Pender, Anne-Louise Ponsonby, Bruce Taylor, Patricia C Valery, Ingrid van der Mei, David Williams

**Affiliations:** Barwon Health, PO Box 281, Geelong, VIC 3220, Australia; National Centre for Epidemiology and Population Health, The Australian National University, Cnr Mills and Eggleston Roads, Canberra 2601, Australia; The Florey Institute of Neuroscience and Mental Health, 30 Royal Pde, Parkville, VIC 3052, Australia; Menzies Institute for Medical Research, University of Tasmania, 17 Liverpool St, Hobart, Australia

**Keywords:** environment, MRI, genetics, multiple sclerosis, conversion

## Abstract

Understanding the predictors of progression from a first to a second demyelinating event (and formerly, a diagnosis of clinically definite multiple sclerosis) is important clinically. Previous studies have focused on predictors within a single domain, e.g. radiological, lacking prospective data across multiple domains. We tested a comprehensive set of personal, environmental, neurological, MRI and genetic characteristics, considered together, as predictors of progression from a first demyelinating event to clinically definite multiple sclerosis. Participants were aged 18–59 years and had a first demyelinating event during the study recruitment period (1 November 2003–31 December 2006) for the Ausimmune Study (*n* = 216) and had follow-up data to 2–3 years post-initial interview. Detailed baseline data were available on a broad range of demographic and environmental factors, MRI, and genetic and viral studies. Follow-up data included confirmation of clinically definite multiple sclerosis (or not) and changes in environmental exposures during the follow-up period. We used multivariable logistic regression and Cox proportional hazards regression modelling to test predictors of, and time to, conversion to clinically definite multiple sclerosis. On review, one participant had an undiagnosed event prior to study recruitment and was excluded (*n* = 215). Data on progression to clinically definite multiple sclerosis were available for 91.2% (*n* = 196); 77% were diagnosed as clinically definite multiple sclerosis at follow-up. Mean (standard deviation) duration of follow-up was 2.7 (0.7) years. The set of predictors retained in the best predictive model for progression from a first demyelinating event to clinically definite multiple sclerosis were as follows: younger age at first demyelinating event [adjusted odds ratio (aOR) = 0.92, 95% confidence interval (CI) = 0.87–0.97, per additional year of age); being a smoker at baseline (versus not) (aOR = 2.55, 95% CI 0.85–7.69); lower sun exposure at age 6–18 years (aOR = 0.86, 95% CI 0.74–1.00, per 100 kJ/m^2^ increment in ultraviolet radiation dose), presence (versus absence) of infratentorial lesions on baseline magnetic resonance imaging (aOR = 7.41, 95% CI 2.08–26.41); and single nucleotide polymorphisms in human leukocyte antigen (*HLA*)*-B* (rs2523393, aOR = 0.25, 95% CI 0.09–0.68, for any G versus A:A), *TNFRSF1A* (rs1800693, aOR = 5.82, 95% CI 2.10–16.12, for any C versus T:T), and a vitamin D-binding protein gene (rs7041, aOR = 3.76, 95% CI 1.41–9.99, for any A versus C:C). The final model explained 36% of the variance. Predictors of more rapid progression to clinically definite multiple sclerosis (Cox proportional hazards regression) were similar. Genetic and magnetic resonance imaging characteristics as well as demographic and environmental factors predicted progression, and more rapid progression, from a first demyelinating event to a second event and clinically definite multiple sclerosis.

## Teaser

Chapman *et al.* report that in a cohort of people with a first demyelinating event, younger age, smoking, low sun exposure, infratentorial lesions on MRI, and genes in HLA, vitamin D-binding protein, and tumour necrosis factor receptor 1A predicted faster progression to clinically definite multiple sclerosis over 2–3 years.

## Graphical Abstract

**Graphical abstract:**
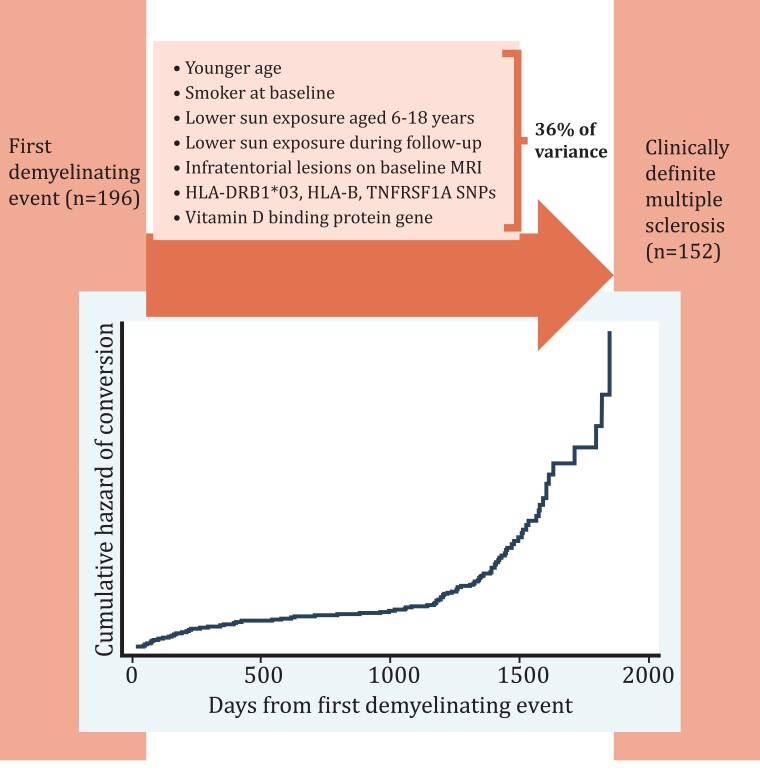

## Introduction

The first episode of clinically eloquent CNS inflammatory demyelination is frequently the first clinical manifestation of multiple sclerosis. This may occur on a background of sub-clinical disease that has been present for many years prior.^1,2^ This first episode is commonly referred to as a first demyelinating event. In the most recent McDonald Criteria, the diagnosis of multiple sclerosis can be made on the basis of a first demyelinating event with MRI lesions and CSF-specific oligoclonal bands, but may often still be made following a second clinical episode.^3^ The period between a first demyelinating event and a second clinical event (and clinical diagnosis of multiple sclerosis) is of significant interest to patients and clinicians as an opportunity for effective treatment to alter the natural history of disease progression.^4^ In addition, there remains a minority of people who suffer a first demyelinating event and never have further episodes or new MRI activity.^5^ Given this heterogeneity in outcome, understanding factors, particularly modifiable factors, that drive early progression to a second event after a first demyelinating event remains of considerable interest.

Many previous studies have examined a typically narrow set of predictors of progression from a first demyelinating event to clinically definite multiple sclerosis in a wide range of populations (recent examples include other studies^6–11^). Such studies have highlighted that older age at first demyelinating event, optic neuritis, isolated sensory symptoms, any exposure to a disease-modifying drug, and normal MRI impart a more favourable prognosis. Alternatively, first symptom location in the brainstem or the supratentorial region (compared to the optic pathways),^12^ having at least one gadolinium-enhancing MRI lesion, and three or more periventricular lesions were factors associated with worse prognosis.^6,9,12,13^

Most studies of progression after a first demyelinating event have involved referral-based clinical samples with often significantly delayed enrolment into the study and potential loss of very early converters, and a focus on clinical or MRI parameters, with few data across the breadth of potential predictors, such as environmental and genetic factors. Few prospective population-based data exist. In recent years, early treatment with disease-modifying therapies may now make it impossible to study predictors of natural disease progression. No previous work has investigated the relationship between progression from a first demyelinating event to clinically definite multiple sclerosis in relation to personal and population-level environmental factors such as residential location, that may be important given the latitudinal gradient of multiple sclerosis that has been described in Australia and elsewhere.^14–16^

Here, we examine factors associated with early progression from a first demyelinating event to a second event (and thus clinically definite multiple sclerosis)—within 3 years of the first demyelinating event—in a well-characterized adult population on which there are detailed environmental, genetic, and radiological data at the time of the first demyelinating event and 2–3 years later. The study participants were recruited from 1 November 2003 to 31 December 2006 in Australia—when therapeutic options for first demyelinating event were largely limited to steroids at the time of the acute event, with subsidized access to disease-modifying therapies requiring a definitive diagnosis of multiple sclerosis, at the time defined by a second clinical event. We hypothesize that there will be a range of demographic, environmental, genetic factors, and MRI parameters that will, together, predict whether and how rapidly adults recruited at the time of their first demyelinating event will be diagnosed with multiple sclerosis within 2–3 years of follow-up.

## Materials and methods

The Ausimmune Study^17^ was a population-based multi-centre incident case–control study designed to capture all incident first demyelinating event cases in four regions of Australia of differing latitude: Brisbane city (latitude 27°S), Newcastle city and surrounds (33°S), Geelong city and the Western Districts of Victoria (37°S) and the island of Tasmania (43°S). The study included follow-up at 2–3 years from baseline for all case participants.

### Participants

Eligible cases were aged 18–59 years, lived within one of the study regions and had a first clinical diagnosis of CNS demyelinating disease during the recruitment period. Details of case ascertainment have been previously reported.^18^

**Figure 1:**
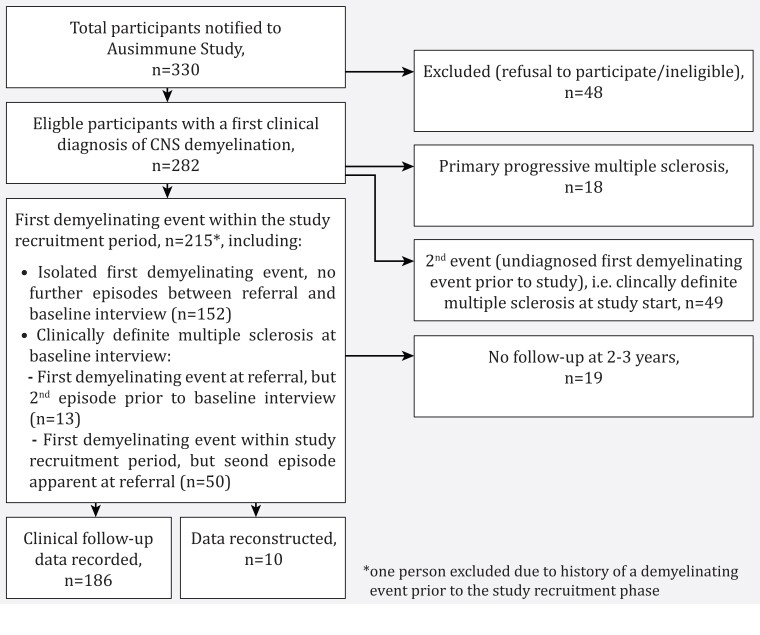
Flow chart of case participation in the Ausimmune Study.

Cases were classified as: first demyelinating event within the period of study recruitment; a second demyelinating event having had a historical, previously undiagnosed, episode recognized at enrolment in the Ausimmune study; or primary progressive multiple sclerosis (see Fig. 1).^18^ This study focuses on participants with a first demyelinating event within the period of study recruitment, further defining the subgroup who, at the baseline interview, had had a first demyelinating event only (i.e. no second event).

### Data collected

#### Baseline only data

Participants self-reported demographic data (age, sex, parity), smoking history, history of infectious mononucleosis (yes/no/do not know),^19^ time outdoors and location for each year of life from age 6 years during weekends and holidays, and physical activity.^20^ At a face-to-face interview, research officers measured height and weight, and made silicone rubber impressions (‘casts’) of the skin on the back of both hands.^21^ Most participants provided a blood sample (96% of cases). Serum aliquots were stored at −80°C and analysed for concentration of 25-hydroxyvitamin D [25(OH)D] at the completion of the baseline study, using liquid chromatography dual mass spectrometry.^18^ DNA was extracted from whole blood; single nucleotide polymorphism (SNP) genotyping for candidate SNPs in the vitamin D pathway (67 SNPs), related to skin colour (17 SNPs), inflammatory pathways (23 SNPs, including tumour necrosis factor receptor superfamily member 1A (*TNFRSF1A*)), and human leukocyte antigen (*HLA*)*-DR15* (rs9271366, rs3135388), *HLA DRB1* (rs2187688, rs660895, rs7755224), *HLA* Class 1 region (rs69040209, rs2854050), and *HLA-B* (rs2523393) was performed using the SNPline method (KBiosciences, Hoddesdon Herts, UK). In the majority of case participants (*n* = 213), quantitative IgG antibody titres to Epstein Barr virus nuclear antigen and human herpes virus 6 were also measured, as previously described.^19^ MRI data were acquired from clinical MRIs undertaken as part of the initial clinical work-up.^22^

#### Follow-up data

Research officers obtained copies of all doctors’ letters and MRI reports since initial participation in the Ausimmune Study. Self-reported questionnaire data included changes in employment status or occupation; recent sun exposure in summer and winter and separately for week days, weekends and holidays, and sunburn; physical activity; and smoking status.

#### Neurological assessment at baseline and follow-up

Study neurologists confirmed symptomatology, date of first demyelinating event onset (baseline only), recent and current medications and, at follow-up, history of relapses (including date, affected systems and clinical management). Conversion was defined clinically up until the final study review at 2–3 years from the baseline interview. At this time point, those who had not converted clinically were offered an MRI scan and on this basis were deemed to have converted if there was evidence of new T2 lesions consistent with demyelination and corroborated by review by a study neuroradiologist.^23^ We defined progression to clinically definite multiple sclerosis as both clinical conversion and meeting the McDonald 2005/McDonald 2010 MRI criteria for multiple sclerosis.^23^ For a subset of participants, follow-up data were reconstructed by a study neurologist from doctors’ letters and MRI scans or reports undertaken during normal clinical review, as MRI and/or full study neurological assessment were unavailable. The purpose was to identify progression to clinically definite multiple sclerosis or not for as many eligible case participants as possible.

### Statistical analysis

*Data management:* We calculated the total years of smoking at baseline, subtracting years of cessation; smoking was modelled both as this continuous variable and as dichotomous smoking status (yes/no). Body mass index was calculated as weight (kg)/height (m^2^). Physical activity was scored according to the International Physical Activity Questionnaire guideline^20^ and categorized as low, medium, high. Cumulative dose of ultraviolet radiation from six-current age was annualized by dividing the total ultraviolet radiation dose by the age minus 6 years, to account for age differences (and thus the opportunity for exposure to ultraviolet radiation). We calculated the ultraviolet radiation dose in the summer and winter prior to the follow-up interview using questionnaire data on time in the sun and the geocoded location of residence to derive average daily erythemally effective ambient ultraviolet radiation dose.^18^ We created two binary variables of low (<50 versus ≥50 nmoL/L) and high (≥75 versus <75 nmoL/L) 25(OH)D based on the total 25(OH)D level. We developed a four-category variable combining smoking (yes/no) at baseline and follow-up as follows: yes/yes; yes/no; no/yes; no/no. We categorized Epstein Barr virus nuclear antigen titres as ≤1:160 (low) versus >1:160 dilution (high) and human herpes virus 6 immunoglobulin G titre as ≤40 (low) and >40 (high). SNPs were coded as binary variables due to small numbers.

*Data analysis:* We used descriptive statistics [mean (standard deviation), median (25th percentile, 75th percentile) or number (proportion)] to characterize the participant sample, as appropriate for the continuous or categorical nature of the data and whether continuous data were normally distributed.

We examined progression to clinically definite multiple sclerosis or not (yes/no) at 2–3 year follow-up according to demographic factors, environmental exposures, baseline first demyelinating event phenotype and symptom topography, MRI characteristics, and genetic factors, using simple logistic regression. We used multiple logistic regression models and backward stepwise regression followed by purposive selection of covariates using likelihood ratio tests to determine the best predictors of progression to clinically definite multiple sclerosis (yes/no) within groups of explanatory variables, e.g. environmental factors, genetic factors, etc., and Cox proportional hazards regression to determine exposures at baseline, and initial and subsequent treatment, as predictors of time to clinically definite multiple sclerosis diagnosis. All logistic regression models were minimally adjusted for time from baseline to follow-up interview. Results are presented as adjusted odds ratios (aORs) or hazard ratios (aHRs) with 95% confidence intervals (CIs) and exact *P*-values. Participants with missing data were excluded from analyses. All statistical tests were two-tailed; *P* < 0.05 was used to determine statistical significance.

Sensitivity analyses were conducted as: (i) confining the main analyses to clinical conversion only, without the addition of the reconstructed neurological assessment data and (ii) in the Cox regression analysis, excluding those with unknown day or month of clinically definite multiple sclerosis diagnosis for date of first demyelinating event (in the main analysis, unknown day is coded as ‘15’ and unknown month as ‘6’).

The Ausimmune Study was approved by nine regional Human Research Ethics Committees. All participants gave written informed consent.

*Data availability:* Access to the Ausimmune Study data in collaboration with the Ausimmune Investigator Group may be available through the authors.

## Results

This study focused on the 216 case participants in the Ausimmune Study who had an incident first demyelinating event within the recruitment period (see Fig. 1).^18^ On further review of clinical notes during this study, one participant was reclassified as having an initial first demyelinating event prior to the study recruitment period (and was thus excluded from this analysis).

Data on diagnosis (or not) of clinically definite multiple sclerosis were available for 91.2% (*n* = 196) of eligible case participants with a first demyelinating event, with reconstructed data constituting 5.1% (*n* = 10) of these. Mean (standard deviation) time from baseline to follow-up interview was 2.7 (0.7) years and from the date of the first demyelinating event to follow-up interview was 3.4 (0.6) years.

### Conversion to multiple sclerosis

Of the 215 participants with a first demyelinating event, 30% (*n* = 63) had had a second demyelinating event by the time of the baseline interview, and were thus already diagnosed as clinically definite multiple sclerosis. Of the *n* = 152 participants with a first demyelinating event only at baseline interview, follow-up data were available on 133 participants, of whom 66.9% (*n* = 89) were diagnosed with clinically definite multiple sclerosis by the 2–3 year follow-up. Thus, of the initial 215 participants who were classified as ‘first demyelinating event within the recruitment period’ at baseline, there was sufficient information (either from follow-up interview/MRI or because they were already clinically definite multiple sclerosis at baseline interview) to make a determination about progression to clinically definite multiple sclerosis by 2–3 years on 196 participants: 77.7% (*n* = 152) had been diagnosed as clinically definite multiple sclerosis. Table 1 shows the distribution of included case participants across study regions. Supplementary Table 1 provides baseline data for all participants with a first demyelinating event within the study recruitment period, and separately, those with a first demyelinating event only at baseline interview. Supplementary Table 2 shows participant and baseline characteristics separately by location of residence.

**Table 1:** Distribution of Ausimmune Study case participants across study regions according to the first demyelinating event and progression to clinically definite multiple sclerosis at baseline and follow-up

	Brisbane (27°S)	Newcastle (33°S)	Geelong (37°S)	Tasmania (43°S)	Overall
Eligible Ausimmune Study case participants, *n* (%)	93 (33.0)	39 (13.8)	70 (24.8)	80 (28.4)	282
First demyelinating event, *n* (%)	67 (31.2)	32 (14.9)	47 (21.9)	69 (32.1)	215
First demyelinating event with data on conversion to clinically definite multiple sclerosis, *n* (%)^a^	57 (89.1)	29 (90.6)	42 (89.4)	68 (98.6)	196 (91.2)
First demyelinating event progressed to clinically definite multiple sclerosis, *n* (%)^a^	45 (79.0)	27 (93.1)	33 (78.6)	47 (69.1)	152 (77.6)

^a^ Includes those with a first demyelinating event within the study recruitment period, but then a second event prior to baseline interview, i.e. clinically definite multiple sclerosis at baseline interview (based on *n* = 196 participants with data available on clinically definite multiple sclerosis diagnosis).

### Progression to clinically definite multiple sclerosis according to demographic and environmental factors

Supplementary Table 3 shows the individual demographic and environmental factors as potential predictors of progression to clinically definite multiple sclerosis by 2–3 years for all participants with a first demyelinating event during study recruitment and separately those with a first demyelinating event only at the baseline interview. Table 2 shows the factors and their effect estimates that were retained as predictors of progression to clinically definite multiple sclerosis in the best predictive models. None of low 25(OH)D, high 25(OH)D, or deseasonalized (continuous) 25(OH)D concentration were significant predictors of progression to clinically definite multiple sclerosis, and none were retained in the predictive model. The models explained 11% of the variance in progression to clinically definite multiple sclerosis. Greater sun exposure on weekends during the summer prior to follow-up was associated with a lower risk of progression to clinically definite multiple sclerosis, but this was not retained in the predictive model due to considerable missing data that would have reduced the sample size of this model.

**Table 2:** Demographic and environmental predictors of progression to clinically definite multiple sclerosis in ‘all first demyelinating event’ and ‘first demyelinating event only at baseline interview’ participants, results from the best predictive model

	All first demyelinating event aOR (95% CI), *P*	First demyelinating event only at baseline interview aOR (95% CI), *P*
Age (per 1-year increment)	0.95 (0.91–0.99) *P = 0.01*	0.94 (0.90–0.99) *P = 0.009*
Current smoker at baseline	2.27 (0.96–5.36) *P = 0.06*	2.60 (1.03–6.59) *P = 0.04*
Leisure time sun exposure from 6 to 18 years, per 100 kJ/m^2^ increment)	0.89 (0.80–0.99) *P = 0.04*	0.87 (0.77–0.99) *P = 0.03*
Pseudo *R*^2^, *P* for the model	0.06, *P = 0.01*	0.11, *P = 0.001*

### Progression to clinically definite multiple sclerosis according to genetic factors

The distribution and minimally adjusted *P*-values for progression to clinically definite multiple sclerosis in relation to measured SNPs are shown in Supplementary Table 4. The results of the best-fitting predictive model of genetic factors are shown in Table 3 (all coded as binary, according to Table 3). This model explained 14% and 15% of the variance for the all first demyelinating event analysis and that for the group with a first demyelinating group only at the baseline interview, respectively.

**Table 3:** Genetic predictors of conversion to multiple sclerosis in ’all first demyelinating event’ and those with a ’first demyelinating event only at the baseline interview’, results from the best predictive model

	All first demyelinating event aOR (95% CI), *P*	First demyelinating event only at baseline interview aOR (95% CI), *P*
*TNFRSF1A* (rs1800693) Any C versus TT	2.82 (1.23–6.50) *P = 0.02*	3.31 (1.31–8.39) *P = 0.01*
Vitamin D-binding protein (rs7041) Any A versus CC	2.44 (1.97–5.58) *P = 0.04*	2.04 (0.83–4.97) *P = 0.12*
Vitamin D receptor (*VDR*) (rs2283342) Any G versus A:A	2.74 (0.93–8.06) *P = 0.07*	2.55 (0.80–8.14) *P = 0.11*
HLA DRB1*03 (rs2187688) Any A versus G:G	2.15 (0.89–5.20) *P = 0.09*	2.12 (0.80–5.62) *P = 0.13*
*HLA-B* (rs2523393) Any G versus A:A	0.47 (0.20–1.10) *P = 0.08*	0.63 (0.25–1.62) *P = 0.34*
Pseudo R^2^, *P* for the model	0.14, *P = 0.0006*	0.15, *P = 0.002*

### Progression to clinically definite multiple sclerosis according to baseline neurological and MRI characteristics

The distributions of all first demyelinating event participants and those with a first demyelinating event only at the baseline interview according to baseline neurological and MRI characteristics are shown in Supplementary Table 5. The results of the best-fitting predictive model for this set of factors are shown in Table 4. The results were similar for number of Barkhof criteria and total T2 lesions in brain in terms of predicting progression to clinically definite multiple sclerosis, but the CIs were much wider for the latter; we thus retained Barkhof criteria in the model by preference.

**Table 4:** Baseline neurological and MRI characteristics as predictors of progression to clinically definite multiple sclerosis in ’all first demyelinating event’ and participants with a ’first demyelinating event only at the baseline interview’, results from the best predictive model

	All first demyelinating event aOR (95% CI), *P*	First demyelinating event only at baseline interview aOR (95% CI), *P*
Presentation with optic neuritis (versus any other phenotype)	0.38 (0.16–0.89) *P = 0.03*	0.26 (0.09–0.74) *P = 0.01*
Number of Barkhof criteria (versus 0)		
1–2 criteria	4.98 (1.62–15.26) *P = 0.005*	12.58 (2.38–66.32) *P = 0.003*
3–4 criteria	4.82 (1.62–14.40) *P = 0.005*	24.93 (2.86–217.34) *P = 0.004*
Presence of infratentorial lesions (y/n)	4.86 (1.32–17.88) *P = 0.02*	5.77 (1.39–23.98) *P = 0.02*
Presence of juxtacortical lesions (y/n)	Not retained in the model	0.20 (0.04–1.06) *P = 0.06*
Pseudo R^2^, *P* for the model	0.17, *P < 0.0001*	0.24, *P = 0.0001*

### Progression to clinically definite multiple sclerosis according to medications and changes in behaviour post-diagnosis

Of all participants with a first demyelinating event, 20.9% (20.6% in those with a ’first demyelinating event only at baseline interview) had received methylprednisolone at baseline, and 30.2% (42.8% in those with a first demyelinating event only at the baseline interview) had received some form of steroid. Neither of these medications were associated with conversion to multiple sclerosis by 2–3 year review in either the ‘all first demyelinating event’ group or those with a first demyelinating event only at the baseline interview (data not shown). Only 6.5% of first demyelinating event participants (9.2% of those with a first demyelinating event only at baseline interview) were on multiple sclerosis disease-modifying therapy (all interferon-beta) at the time of the baseline interview—all were diagnosed as clinically definite multiple sclerosis by the 2–3 year follow-up.

At baseline, 22.7% of all first demyelinating event (32.2% of those with a first demyelinating event only at baseline interview) participants were taking a vitamin D-containing supplement, usually a multivitamin. By the 2–3 year review, 22.7% of first demyelinating event participants (but not all of the same participants) (24.1% of in those with a first demyelinating event only at baseline interview) were taking a vitamin D supplement. No association was evident between vitamin D supplementation (yes/no) or dose at baseline and progression to clinically definite multiple sclerosis (*P* = 0.27, *P* = 0.81, respectively), but these results are based on small numbers in the exposed groups.

In participants with a first demyelinating event only at baseline interview, higher summer weekend or holiday sun exposure reported at the follow-up interview (both aOR = 0.72, 95% CI 0.53–0.97, *P* = 0.03 per greater hour of sun exposure), and higher dose of ultraviolet radiation^18^ (aOR = 0.54, 95% CI 0.30–0.98, *P* = 0.04 per higher kJ/m^2^) were associated with a lower risk of progression to clinically definite multiple sclerosis. Being a smoker at baseline and follow-up (compared to being a non-smoker at both time-points) was associated with a greater risk of progression to clinically definite multiple sclerosis by 2–3 year follow-up (in those with a first demyelinating event only at baseline interview, aOR = 2.90, 95% CI 1.07–7.87). Change in smoking status was not associated with risk of conversion, but, this analysis was based on a very small number of people who became smokers (*n* = 5) or stopped smoking (*n* = 6).

### Overall predictive model of progression to clinically definite multiple sclerosis (or not) by 2–3 years of review

The results of the overall predictive model are shown in Table 5. Consistent across ‘all first demyelinating event’ and those with a first demyelinating event only at baseline interview, older age at baseline, higher past sun exposure, and an *HLA-B* SNP were associated with lower risk of progression to clinically definite multiple sclerosis, while SNPs within the vitamin D-binding protein gene and *TNFRSF1A*, and the presence of infratentorial lesions on baseline MRI, were associated with increased risk. Supplementary Table 6 shows the results of the overall predictive model excluding from the analysis those who had follow-up data reconstructed from clinical notes.

**Table 5:** Results of the best predictive logistic regression model of the predictors of progression to clinically definite multiple sclerosis within 2-3 years following a first demyelinating event

	All first demyelinating event (*n* = 157)		First demyelinating event only at baseline interview (*n* = 109)	
Variable	aOR (95% CI)	*P*	aOR (95% CI)	*P*
Age at baseline (per 1 year increment)	0.92 (0.87–0.97)	*0*.*003*	0.90 (0.85–0.97)	*0*.*002*
Current smoker at baseline (yes, versus no)	2.56 (0.85–7.71)	*0*.*09*	3.87 (1.12–13.33)	*0*.*03*
Leisure time sun exposure from 6–18 years (per 100 kJ/m^2^ increment)	0.87 (0.75–1.01)	*0*.*06*	0.87 (0.73–1.03)	*0*.*11*
*HLA-B* (rs2523393) Any G versus A:A	0.26 (0.10–0.72)	*0*.*009*	0.29 (0.09–0.93)	*0*.*04*
*TNFRS1A* (rs1800693) Any C versus T:T	5.76 (2.08–15.93)	*0*.*001*	7.61 (2.24–25.91)	*0*.*001*
Vitamin D-binding protein (rs7041) Any A versus C:C	3.49 (1.30–9.34)	*0*.*01*	2.59 (0.85–7.85)	*0*.*09*
Infratentorial lesions on MRI (yes versus no)	7.37 (2.06–26.41)	*0*.*002*	7.52 (1.80–31.37)	*0*.*006*
Pseudo R^2^, *P* for the model	0.32, *P < 0.0001*		0.36, *P < 0.0001*	

### Time to clinically definite multiple sclerosis diagnosis

Supplementary Table 7 shows the minimally adjusted results of the Cox proportional hazards regression models for each individual variable. The results of the best-fitting overall predictive model for all first demyelinating event participants are shown in Table 6. Number of Barkhof criteria and total number of T2 lesions on the baseline MRI were not retained in the model.

**Table 6:** Results of the best-fitting Cox proportional hazards regression model for progression from a first demyelinating event to clinically definite multiple sclerosis by the follow-up interview in all participants (*n* = 166)

Variable	aHR (95% CI)	*P*
Age at baseline (per 1 year increment)	0.98 (0.96–1.00)	*0*.*02*
Leisure time sun exposure from 6–18 years (per 100 kJ/m^2^ increment)	0.92 (0.87–0.98)	*0*.*007*
*TNFRS1A* (rs1800693) Any C versus T:T	1.57 (1.05–2.34)	*0*.*03*
Steroid treatment (yes versus no)	1.72 (1.14–2.58)	*0*.*009*
*P* (model)		*0*.*0001*

Supplementary Table 7 also shows the results of the Cox proportional hazards regression model restricting the analysis to participants with no imputed day of month or month of year data for the first demyelinating event or multiple sclerosis diagnosis. The results were very similar to those for the main analysis.

## Discussion

This study provides significant and clinically relevant information on multiple factors that predict early progression to clinically definite multiple sclerosis (typically a second event) after a first demyelinating event. While previous studies have examined single risk factors or multiple risk factors within a single domain, e.g. environmental, demographic, genetic, MRI, here we built a single model across multiple domains to identify significant predictors after mutual adjustment. Furthermore, the study provides unique data for the 3 years after a first demyelinating event—a potentially important period that most studies are not designed to evaluate.

In the overall predictive model, the most important predictors of progression to clinically definite multiple sclerosis were younger age, low past sun exposure, genetic factors including HLA and vitamin D-related SNPs, and baseline MRI characteristics. In minimally adjusted models, the risk of progression to clinically definite multiple sclerosis increased by 67% for each category increase in T2 lesions (Categories 1–3, 4–6, 7–9, and >9, compared to 0), and this was mirrored by a similar increase in the rate of conversion with each additional Barkof criterion met. However, neither of these measures was retained in the overall predictive model, with the presence of infratentorial lesions being most important. Similarly, although conversion was less likely with an optic neuritis presenting phenotype, this was not retained in the overall predictive model. We did not see any association with latitude in this study, suggesting that the factors that drive the latitudinal gradient of incident first demyelinating event and multiple sclerosis^15^ may be distinct from those that drive progression to clinically definite multiple sclerosis.

Environmental factors that have been associated with multiple sclerosis risk have not been frequently studied for their association with progression to clinically definite multiple sclerosis. One study in 211 patients with a first demyelinating event and abnormal MRI scan found that risk of progression was associated with higher Epstein Barr virus viral capsid antigen immunoglobulin levels, and relapses with higher levels of antibodies to cytomegalovirus, with no association with serum 25(OH)D levels.^24^ Here we found no association with Epstein Barr virus nuclear antigen or human herpes virus 6 antibody levels or 25(OH)D levels, but higher risk and greater hazard of progression to clinically definite multiple sclerosis in association with smoking and lower past sun exposure, for the latter both at baseline and as reported at follow-up. These findings are consistent with sun exposure providing longer-term immunological benefits rather than short term effects as indicated by the 25(OH)D level. Previous studies have also indicated a link between higher sun exposure and better outcomes in relapsing remitting multiple sclerosis,^25,26^ but have been unable to rule out reverse causality. Our previously published follow-up analysis focused on environmental risk factors^27^ shows that this protective effect of higher sun exposure persists to 5 years from baseline.

Genetic markers of progression in multiple sclerosis have not been well described. Surprisingly, considering its substantial association with multiple sclerosis risk, there is little evidence to suggest that HLA DRB1*1501 is associated with multiple sclerosis progression^28,29^ and this is consistent with our findings here. We did however find SNPs in *HLA-B* and the vitamin D-binding protein were significant predictors of risk of progression to clinically definite multiple sclerosis. The latter finding may reflect lower bioavailable 25(OH)D and/or effects on the serum half-life of 25(OH)D associated with this polymorphism.^30^ Despite its name, the vitamin D-binding protein is multifunctional and conserved through vertebrate evolution.^31^ In addition to binding all of the vitamin D metabolites, it binds to membrane receptors controlling epithelial absorption, including in the brain; actin, acting as an actin-scavenger; and fatty acids (particularly polyunsaturated fatty acids) and membrane phospholipids of activated neutrophils leading to enhanced complement chemotactic activity.^31^ Several previous studies (reviewed in ^32^) have linked vitamin D-binding protein to multiple sclerosis, but causal pathways remain to be elucidated.

In this study we found that the functional SNP rs1800693 in the *TNFRSF1A* gene, encoding tumour necrosis factor receptor superfamily member 1A,^33,34^ was strongly predictive of progression to clinically definite multiple sclerosis: those carrying the C allele had a nearly 6-fold higher risk. The C allele is associated with the production of a novel soluble form of the tumour necrosis factor alpha receptor that binds tumour necrosis factor alpha and reproduces the effects of tumour necrosis factor alpha blockers,^33^ that may induce or exacerbate CNS demyelination.^35^ We have previously shown in a longitudinal study that the protective effect of higher tumour necrosis factor alpha levels (in peripheral blood mononuclear cells) for multiple sclerosis relapse was apparent only in those carrying the CC allele of the *TNFRSF1A* gene.^34^ Thus, the functional consequences of this SNP may relate to interactions with other genes and cytokines.^34^

Here, we found that methylprednisolone or other steroid therapy at baseline did not affect the risk of progression to clinically definite multiple sclerosis but was associated with a greater hazard of progression. The latter could reflect that steroids are given for more severe disease at onset (e.g. greater inflammation) and more rapid progression to clinically definite multiple sclerosis. A lack of benefit in relation to progression to clinically definite multiple sclerosis following optic neuritis has been previously reported,^36,37^ while another study showed steroids to be effective in exacerbations, but not in preventing disease progression in multiple sclerosis.^38^

Higher serum 25(OH)D at baseline was not a significant predictor of either progression or time to clinically definite multiple sclerosis. Of note, only a small proportion of participants were taking even low dose vitamin D supplementation in this study, so that we were unable to properly study whether vitamin D supplementation post-first demyelinating event altered progression to clinically definite multiple sclerosis or time to clinically definite multiple sclerosis diagnosis. However, the findings of protective associations for higher ultraviolet radiation dose over the lifetime or in early life, and of higher sun exposure in the summer prior to the follow-up interview, as well as the links between vitamin D-related genes and multiple sclerosis, support recent results of a possible protective effect of ultraviolet-B phototherapy,^39^ and/or vitamin D supplementation.

These findings provide significant information on the risk of early progression to clinically definite multiple sclerosis in a relatively large population-based first demyelinating event cohort living across a diverse latitudinal range. They emphasize the importance of modifiable risk factors such as smoking and lifetime sun exposure, but also of genetic factors. The study has some limitations. It included participants ranging in age from 18 to 59 years. The accuracy and precision of the recall of early life exposures may vary according to age, thus potentially introducing bias into the study. In addition, we were unable to assess some potential risk factors, such as new pregnancies during the follow-up period, due to small numbers. Perhaps surprisingly, our final predictive models did not include major well-known risk factors for multiple sclerosis onset such as Epstein Barr positivity or titre^40^ and HLA DRB1*1501. This is consistent with previous studies^29,41^ and may relate to features of study design rather than truly suggesting that risk factors for onset differ to those of progression. Risk factors for onset typically use a case–control or nested case–control design; the comparison groups are people with and without the disease, and the study design is ideally suited to an uncommon disease such as multiple sclerosis, with the ability to increase study power by increasing the size of the control/unaffected group. In studying predictors within a group of people with multiple sclerosis or a first demyelinating event, there is both less difference between the comparison groups (rather than having or not having the disease, there are shades of more or less active disease) and typically a smaller sample size. Our understanding of the pathology of multiple sclerosis would suggest that ‘onset’ is simply the first recognized/clinical event and the risk factors for that expression of the underlying pathology will be the same as for the next and subsequent events.

It is important to note that the data collection occurred prior to the application of the McDonald criteria 2010 and the more recent McDonald criteria 2018 which allow for the diagnosis of multiple sclerosis at the time of the initial first demyelinating event with the aid of MRI,^23^ and CSF oligoclonal bands as well as MRI.^3^ In this cohort it is difficult to retrospectively employ these criteria as the use of gadolinium was limited in the initial MRI scans and lumbar puncture was not a routine part of the work-up for multiple sclerosis in many areas and therefore these diagnostic methods were not evenly nor universally applied. We cannot comment, therefore, on whether we would have been able to diagnose multiple sclerosis earlier in this cohort although it is likely to be the case. It is of relevance to note that only 15 of the 202 participants with data available on their baseline MRI had a normal scan, suggesting that the vast majority could have had multiple sclerosis diagnosed at this time point with either the use of gadolinium or the measurement of oligoclonal bands. This is particularly relevant for the participants who had had a first demyelinating event only at the baseline interview and who had not clinically or radiologically converted at 2–3 years. Nevertheless, a value of the current study is that it provides information on natural—disease-modifying therapy-free—disease progression in relation to a wide range of risk factors, taken together, that may no longer be possible with the broad range of treatment options and aggressive treatment now available for new-onset multiple sclerosis.

## Supplementary Material

fcac181_Supplementary_Data

## Appendix I

The Ausimmune investigator group includes Dr Caron Chapman; Prof Alan Coulthard; Prof Keith Dear; Prof Terry Dwyer; Prof Trevor Kilpatrick; Prof Robyn Lucas; Prof Tony McMichael (dec); Prof Michael *P* Pender; Prof Anne-Louise Ponsonby; Prof Bruce Taylor; Prof Patricia C Valery; A/Prof Ingrid van der Mei and Dr David Williams.

## Abbreviations

25(OH)D =: 25-hydroxyvitamin D
aOR =: adjusted odds ratio
aHR =: adjusted hazard ratio
CI =: confidence interval
*HLA* =: human leukocyte antigen
*P* =: probability
SNP =: single nucleotide polymorphism
*TNFRSF1A* =: tumour necrosis factor receptor superfamily member 1A gene
