# Supplementary material for: Predictors of progression from a first demyelinating event to clinically definite multiple sclerosis
Source: Brain Commun. 2022 Jul 9;4(4):fcac181. doi: 10.1093/braincomms/fcac181 (PMC9308470; doi:10.1093/braincomms/fcac181)
Supplement: fcac181_Supplementary_Data [file fcac181_supplementary_data.zip › Supplementary file_Ausimmune Investigator Group.docx]

**Appendix**

**The Ausimmune Investigator Group includes:**

Dr Caron Chapman (Barwon Health), Prof Alan Coulthard (University of Queensland); Prof Keith Dear (University of Adelaide); Prof Terry Dwyer (Murdoch Childrens Research Institute); Prof Trevor Kilpatrick (Florey Institute for Neuroscience); Prof Robyn Lucas (Australian National University); Prof Tony McMichael (dec, Australian National University); Prof Michael P Pender (University of Queensland); Prof Anne-Louise Ponsonby (Florey Institute for Neuroscience); Prof Bruce Taylor (Menzies Research Institute Tasmania); Prof Patricia C Valery (QIMR Berghofer Institute); A/Prof Ingrid van der Mei (Menzies Research Institute Tasmania); Dr David Williams (Hunter New England Health)
