# Supplementary material for: Predictors of progression from a first demyelinating event to clinically definite multiple sclerosis
Source: Brain Commun. 2022 Jul 9;4(4):fcac181. doi: 10.1093/braincomms/fcac181 (PMC9308470; doi:10.1093/braincomms/fcac181)
Supplement: fcac181_Supplementary_Data [file fcac181_supplementary_data.zip › Supplementary_material.docx]

**Supplementary Table 1** Characteristics of study participants including all diagnosed with a first demyelinating event within the study recruitment period, and those with a first demyelinating event only (i.e. no second event) at the baseline interview

|  | **All first demyelinating event during study recruitment (n=215)** | **First demyelinating event only at baseline interview**  **(n=152)** |
| --- | --- | --- |
| **Mean (SD)** |  |  |
| Age (years) | 37.7 (9.5) | 37.3 (9.6) |
| Serum 25(OH)D (mean(SD)) | 76.6 (32.0) | 74.4 (31.9) |
| **Median (IQR)** |  |  |
| Annualised cumulative ultraviolet radiation dose (100kJ/m^2^) median (25^th^ percentile, 75^th^ percentile) | 0.67  (0.53, 0.83) | 0.66  (0.54, 0.82) |
| Ultraviolet radiation dose 6-18 years (100kJ/m^2^) median (25^th^ percentile, 75 percentile) | 9.61  (7.56, 12.01) | 9.64  (7.53, 11.92) |
| Daily time outdoors in the summer prior to follow-up (hours)  Weekdays  Weekends  Holidays | 1.5 (0.5, 2.5)  2.5 (1.5, 3.5)  2.5 (1.5, 4.5) | 1.5 (0.5, 2.5)  2.5 (1.5, 4.5)  2.5 (1.5, 4.5) |
| Daily time outdoors in the winter prior to follow-up (hours)  Weekdays  Weekends  Holidays | 1.5 (0.4, 1.5)  1.5 (1.5, 2.5)  1.5 (1.5, 3.5) | 0.5 (0.3, 1.5)  1.5 (1.5, 2.5)  1.5 (1.5, 3.5) |
| Ultraviolet radiation leisure dose summer prior to follow-up (100J/m^2^) | 14.86 (9.49, 22.77) | 14.26 (9.31, 23.00) |
| Ultraviolet radiation leisure dose winter prior to follow-up (100J/m^2^) | 2.94 (1.30, 4.52) | 2.50 (1.26, 4.48) |
| Time from FDE to baseline interview (days) | 183 (92, 341) | 138.5 (69.5, 228.5) |
| EDSS at baseline interview | 1 (0, 2) | 1 (0, 2) |
| **n (%)** |  |  |
| Sex (female) | 164 (76.3) | 115 (75.7) |
| Education  Year 10 or less  Year 12 and TAFE  University | 53 (25.0)  108 (50.9)  51 (24.1) | 37 (24.5)  81 (53.6)  33 (21.9) |
| Study region  Brisbane  Newcastle and surrounds  Geelong and surrounds  Tasmania | 67 (31.2)  32 (14.9)  47 (21.9)  69 (32.1) | 46 (30.3)  21 (13.8)  30 (19.7)  55 (36.2) |
| Current Smoker (n/N, %) | 61/212 (28.8) | 44/150 (29.3) |
| Parity (women only)  0  1-2  3+ | 67 (41.1)  70 (42.9)  26 (16.0) | N=115  51 (44.4)  49 (42.6)  15 (13.0) |
| Past history of infectious mononucleosis  No  Yes  Don’t know | 142 (67.0)  56 (26.4)  14 (6.6) | N=150  103 (68.7)  39 (26.0)  8 (5.3) |
| Season of birth  Summer  Autumn/Fall  Winter  Spring | 51 (23.7)  55 (25.6)  58 (27.0)  51 (23.7) | 32 (21.1)  40 (26.3)  43 (28.3)  37 (24.3) |
| Season of onset of first demyelinating event  Summer  Autumn/Fall  Winter  Spring | 66 (30.7)  52 (24.2)  46 (21.4)  51 (23.7) | 41 (27.0)  41 (27.0)  32 (21.1)  38 (25.0) |
| Cast score  <4  4+ | 90 (43.1)  119 (56.9) | 66 (44.6)  82 (55.4) |
| Epstein Barr virus nuclear antigen immunoglobulin  Low (titre <1:160)  Medium (titre=1:160)  High (titre>1:160) | N=163  35 (21.5)  75 (46.0)  53 (32.5) | N=114  19 (16.7)  56 (49.1)  39 (34.2) |
| Human herpes virus 6 immunoglobulin  Low (titre ≤1:40)  High (titre >1:40) | N=162  52 (32.1)  110 (67.9) | N=114  38 (33.3)  76 (66.7) |

**Supplementary Table 2** Participant and baseline disease characteristics in participants with a first demyelinating event only (i.e. no second event) at the baseline interview, separately by location of residence

|  | **Brisbane** (27°South) | **Newcastle**  (33°South) | **Geelong**  (37°South) | **Tasmania**  (43°South) | **Overall** |
| --- | --- | --- | --- | --- | --- |
| **Age** (mean(SD))  Converted to MS  Not converted | 36.3 (9.0)  38.1 (11.6)  p=0.61 | 34.1 (8.8)  38.7 (27.5)  p=0.59 | 35.1 (11.4)  39.6 (8.8)  p=0.32 | 38.1 (8.7)  43.2 (8.8)  p=0.04 | 36.3 (9.3)  40.9 (10.4)  p=0.01 |
| **Sex**  (n converted/N, %)  Female  Male | 20/31 (64.5)  4/5 (80.0)  p^=0.65 | 12/14 (85.7)  4/4 (100.0)  p^=0.60 | 12/17 (70.6)  4/8 (50.0)  p^=0.29 | 25/38 (65.8)  8/16 (50.0)  p^=0.22 | 69/100 (69.0)  20/33 (60.6)  p=0.37 |
| **Education**  (n converted/N, %)  Year 10 or less  Year 12 and TAFE  University | 7/10 (70.0)  13/19 (68.4)  4/7 (57.1)  p^=0.80 | 3/3 (100.0)  12/13 (92.3)  1/1 (100.0)  p^=1.00 | 3/7 (42.9)  8/11 (72.7)  5/7 (71.4)  p^=0.56 | 9/15 (60.0)  18/27 (66.7)  6/12 (50.0)  p^=0.62 | 22/35 (62.9)  51/70 (72.9)  16/27 (59.3)  p=0.35 |
| **CIS phenotype**  (n converted/N, %)  Optic neuritis  Brainstem/cerebellar  Spinal cord syndrome  Non-visual sensory  Motor  Other | 3/8 (37.5)  7/9 (77.8)  11/16 (68.8)  -  -  3/3 (100.0)  p^=0.20 | 4/4 (100.0)  4/4 (100.0)  5/6 (83.3)  1/1 (100.0)  -  2/2 (100.0)  p^=1.00 | 3/7 (42.9)  4/4 (100.0)  7/9 (77.8)  1/3 (33.3)  -  1/1 (100.0)  p^=0.20 | 10/17 (58.8)  11/15 (73.3)  5/11 (45.5)  1/1 (100.0)  1/1 (100.0)  5/9 (55.6)  p^=0.71 | 20/36 (55.6)  26/32 (81.3)  28/42 (66.7)  3/5 (60.0)  1/1 (100.0)  11/15 (73.3)  p=0.27 |
| **Total T2 lesions on baseline MRI**  (n converted/N, %)  0  1-9  >9 | 0/1 (0.0)  13/18 (72.2)  9/13 (69.2)  p^=0.48 | 0/1 (0.0)  9/9 (100.0)  7/8 (87.5)  p^=0.05 | 0/1 (0.0)  8/12 (66.7)  8/12 (66.7)  p^=0.62 | 1/4 (25.0)  10/18 (55.6)  22/30 (73.3)  p^=0.11 | 1/7 (14.3)  40/57 (70.2)  46/63 (73.0)  p=0.006 |
| **Lesion topography at baseline MRI***  (n converted/N, %)  Infratentorial  Juxtacortical  Periventricular | 4/5 (80.0)  p**=0.49  14/21 (66.7)  p**=0.60  19/27 (70.4)  p**=0.51 | 4/4 (100.0)  p**=0.60  6/7 (85.7)  p**=0.64  14/15 (93.3)  p**=0.31 | 10/12 (83.3)  p**=0.06  12/19 (63.2)  p**=0.64  16/24 (66.7)  p=0.36 | 18/19 (94.7)  p**<0.001  23/31 (74.2)  p**=0.05  32/48 (66.7)  p**=0.13 | 36/40 (90.0)  p<0.001  55/78 (70.5)  p=0.44  81/114 (71.1)  p**=0.07 |

*numbers are progression to clinically definite multiple sclerosis among those who have lesions present in the specific brain location; p values are for comparison of any vs. no lesions in that location and progression to clinically definite multiple sclerosis; non-marked p-values are from chi-square or anova tests

**Fisher exact test 1-sided

^Fisher exact test 2-sided

**Supplementary Table 3** Demographic and environmental factors at baseline according to progression to clinically definite multiple sclerosis or not at 2-3 years follow-up in all participants with a first demyelinating event, and participants with a first demyelinating event only at the baseline interivew (note that all ‘not converted’ participants are by definition in the latter group)

|  | **All first demyelinating event within study recruitment period** | | | **First demyelinating event only at baseline interview** | |
| --- | --- | --- | --- | --- | --- |
| **Clinically definite multiple sclerosis diagnosed at follow-up** | **No** | **Yes** | **p** | **Yes** | **p** |
| **Mean (standard deviation)** |  |  |  |  |  |
| Age, years | 40.9 (10.4) | 37.3 (9.3) | 0.02 | 36.3 (9.3) | 0.01 |
| Total 25(OH)D (nmol/L) | 79.3 (31.7) | 76.7 (31.7) | 0.32 | 73.1 (31.1) | 0.36 |
| **Median (25^th^ percentile, 75^th^ percentile)** |  |  |  |  |  |
| Annualised cumulative ultraviolet radiation dose (100kJ/m^2^) | 0.75 (0.61, 0.87) | 0.64 (0.51, 0.80) | 0.06 | 0.62 (0.51, 0.77) | 0.02 |
| Ultraviolet radiation dose 6-18 years (100kJ/m^2^) | 10.1 (8.4, 12.3) | 9.4 (7.5, 11.7) | 0.06 | 9.3 (7.0, 10.6) | 0.03 |
| Daily time outdoors in the summer prior to follow-up (hours)  Weekdays  Weekends  Holidays | 1.5 (0.5, 2.5)  2.5 (1.5, 4.5)  2.5 (2.5, 4.5) | 1.5 (0.5, 2.5)  2.5 (1.5, 3.5)  2.5 (1.5, 4.5) | 0.44  0.06  0.14 | 1.5 (0.5, 2.5)  2.5 (1.5, 3.5)  2.5 (1.5, 4.5) | 0.47  0.05  0.05 |
| Daily time outdoors in the winter prior to follow-up (hours)  Weekdays  Weekends  Holidays | 1.5 (0.5, 1.5)  1.5 (1.5, 2.5)  2.5 (1.5, 3.5) | 1.5 (0.3, 1.5)  1.5 (1.5, 2.5)  1.5 (1.5, 3.5) | 0.46  0.14  0.20 | 0.5 (0.3, 1.5)  1.5 (0.5, 2.5)  1.5 (0.5, 3.5) | 0.35  0.10  0.09 |
| Ultraviolet radiation dose summer prior to follow-up (100J/m^2^) | 15.7 (12.2, 23.3) | 15.1 (9.4, 21.8) | 0.16 | 14.3 (7.7, 22.7) | 0.07 |
| Ultraviolet radiation dose winter prior to follow-up (100J/m^2^) | 2.9 (1.4, 4.5) | 2.9 (1.3, 4.5) | 0.75 | 2.1 (1.2, 4.3) | 0.86 |
| **n (%)** |  |  |  |  |  |
| Sex  Male  Female | 13 (29.6)  31 (70.5) | 34 (22.4)  118 (77.6) | 0.34 | 20 (22.5)  69 (77.5) | 0.24 |
| Education  Year 10 or less  Year 12 or TAFE*  University | 13 (30.2)  19 (44.2)  11 (25.6) | 38 (25.3)  78 (52.0)  34 (22.7) | 0.34  0.82 | 22 (24.7)  51 (57.3)  16 (18.0) | 0.18  0.86 |
| Study region  Brisbane  Newcastle  Geelong  Tasmania | 12 (27.3)  2 (4.6)  9 (20.5)  21 (47.7) | 45 (29.6)  27 (17.8)  33 (21.7)  47 (30.9) | 0.12  0.99  0.31 | 24 (27.0)  16 (18.0)  16 (18.0)  33 (37.1) | 0.11  0.58  0.49 |
| Current smoker  No  Yes | 34 (79.1)  9 (20.9) | 101 (67.3)  49 (32.7) | 0.17 | 56 (63.6)  32 (36.4) | 0.11 |
| Parity  0  1-2  3+ | 11 (35.5)  15 (48.4)  5 (16.1) | 48 (41.0)  50 (42.7)  19 (16.2) | 0.35  0.61 | 32 (46.4)  29 (42.0)  8 (11.6) | 0.30  0.30 |
| Past history of infectious mononucleosis  No  Yes  Don’t know | 31 (72.1)  8 (18.6)  4 (9.3) | 98 (65.3)  42 (28.0)  10 (6.7) | 0.32  0.82 | 59 (67.1)  25 (28.4)  4 (4.6) | 0.32  0.56 |
| Season of birth  Summer  Autumn  Winter  Spring | 7 (15.9)  9 (20.5)  16 (36.4)  12 (27.3) | 41 (27.0)  38 (25.0)  40 (26.3)  33 (21.7) | 0.91  0.14  0.27 | 22 (24.7)  23 (25.8)  25 (28.1)  19 (21.4) | 0.90  0.15  0.17 |
| Season of onset of first demyelinating event  Summer  Autumn  Winter  Spring | 13 (29.6)  9 (20.5)  8 (18.2)  14 (31.8) | 47 (30.9)  36 (23.7)  35 (23.0)  34 (22.4) | 0.66  0.69  0.36 | 22 (24.7)  25 (28.1)  21 (23.6)  21 (23.6) | 0.28  0.43  0.87 |
| Cast score  <4  ≥4 | 14 (33.3)  28 (66.7) | 65 (43.6)  84 (56.4) | 0.12 | 41 (46.6)  47 (53.4) | 0.09 |
| Epstein Barr virus nuclear antigen immunoglobulin  Low (titre <1:160)  Medium (titre=1:160)  High (titre>1:160) | 8 (22.9)  19 (54.3)  8 (22.9) | 26 (21.9)  52 (43.7)  41 (34.5) | 0.59  0.52 | 10 (14.3)  33 (47.1)  27 (38.6) | 0.66  0.14 |
| Human herpes virus 6 immunoglobulin  Low (titre≤1:40)  High (titre>1:40) | 16 (45.7)  19 (54.3) | 34 (28.8)  84 (71.2) | 0.12 | 20 (28.6)  50 (71.4) | 0.08 |

p-values are derived from logistic regression models with adjustment for time from interview 1 (baseline) to interview 2 (face to face follow-up); TAFE=Technical and Further Education (i.e. trade qualification)

**Supplementary Table 4** Genetic factors in relation to progression to clinically definite multiple sclerosis for all participants with a first demyelinating event and those with a first demyelinating event only at the baseline interview

| **All n (%)** | **All first demyelinating event** | | | **First demyelinating event only at baseline interview** | |
| --- | --- | --- | --- | --- | --- |
| **Diagnosed as clinically definite multiple sclerosis at follow-up** | **No** | **Yes** | **p** | **Yes** | **p** |
| *HLA-DR 15* (rs9271366)  A:A  Any G | 18 (46.2)  21 (53.9) | 58 (42.7)  78 (57.4) | *0.87* | 32 (40.5)  47 (59.5) | *0.39* |
| *HLA DRB1*03* (rs2187688)  G:G  Any A | 15 (37.5)  25 (62.5) | 26 (19.6)  107 (80.5) | *0.05* | 16 (20.5)  62 (79.5) | *0.06* |
| *HLA-B* (rs2523393)  A:A  Any G | 11 (27.5)  29 (72.5) | 60 (44.4)  75 (55.6) | *0.04* | 33 (41.8)  46 (58.2) | *0.20* |
| Vitamin D receptor (*VDR*)  (rs2239186)  T:T  Any C  (rs2283342)  A:A  Any G | 31 (77.5)  9 (22.5)  33 (86.8)  5 (13.2) | 83 (61.0)  53 (39.0)  94 (60.7)  39 (29.3) | *0.02*  *0.04* | 44 (55.7)  35 (44.3)  54 (69.2)  24 (30.8) | *0.01*  *0.05* |
| Vitamin D binding protein (*GC*)  (rs842999)  C:C  Any G  (rs7041)  C:C  Any A | 19 (50.0)  19 (50.0)  20 (50.0)  20 (50.0) | 34 (26.6)  94 (73.4)  34 (25.6)  99 (74.4) | *0.03*  *0.006* | 22 (28.6)  55 (71.4)  21 (27.3)  56 (72.7) | *0.16*  *0.04* |
| *TNFRSF1A* (rs1800693)  T:T  Any C | 19 (47.5)  21 (52.5) | 34 (25.2)  101 (74.8) | *0.007* | 15 (19.2)  63 (80.8) | *0.003* |

None of 18 *CYP24* SNPs, 6 *CYP27* SNPs, 12 *TYR* SNPs, 4 *MC1R* SNPs, 5 *CTLA4* SNPs, 2 *CD58* SNPs, 2 *EV15* SNPs, 4 *IL2RA* SNPs, 1 *CD40* SNP, 1 *IRF8* SNP, or 1 *CD6* SNP were significantly associated with conversion to CDMS (all p>0.05). All SNPs are coded as binary variables due to small numbers.

**Supplementary Table 5**. Neurological and MRI factors as predictors of progression to clinically definite multiple sclerosis in all participants with a first demyelinating event and those with a first demyelinating event only at the baseline interview

| **All n (%)** | **All first demyelinating event** | | | **First demyelinating event only at baseline interview** | |
| --- | --- | --- | --- | --- | --- |
| **Diagnosed as clinically definite multiple sclerosis at follow-up** | **No** | **Yes** | **p** | **Yes** | **p** |
| **Presenting phenotype**  Optic neuritis  Brainstem/cerebellar^#^  Spinal cord syndrome^#^  Non-visual sensory^#^  Motor^#^  Other^#^ | 16 (38.1)  6 (14.3)  14 (33.3)  2 (4.8)  0 (0.0)  4 (9.5) | 30 (20.0)  45 (30.0)  48 (32.0)  5 (3.3)  3 (2.0)  19 (12.7) | 0.006  0.20  0.66  -  0.15 | 20 (22.5)  26 (29.2)  28 (31.5)  3 (3.4)  1 (1.1)  11 (12.4) | 0.006  0.23  0.67  -  0.15 |
| **Total T2 lesions on baseline MRI**  0  1-9  >9 | 6 (15.0)  17 (42.5)  17 (42.5) | 1 (0.7)  63 (42.9)  83 (56.5) | 0.005  0.002 | 1 (1.2)  40 (46.0)  46 (52.9) | 0.02  0.009 |
| **Lesion topography on baseline MRI ***  Infratentorial  No  Yes  Juxtacortical  No  Yes  Periventricular  No  Yes | 36 (90.0)  4 (10.0)  17 (42.5)  23 (57.5)  7 (17.5)  33 (82.5) | 85 (57.8)  62 (42.2)  39 (27.1)  105 (72.9)  10 (6.8)  138 (93.2) | 0.001  0.06  0.05 | 51 (58.6)  36 (41.4)  30 (35.3)  55 (64.7)  6 (6.9)  81 (93.1) | 0.001  0.30  0.05 |
| **No. Barkhof criteria on baseline MRI**  **0**  **1**  **2**  **3**  **4** | 13 (32.5)  8 (20.0)  3 (7.5)  15 (37.5)  1 (2.5) | 11 (7.6)  21 (14.6)  23 (16.0)  52 (36.1)  37 (25.7) | 0.03  0.002  0.004  <0.001 | 8 (9.4)  13 (15.3)  15 (17.7)  30 (35.3)  19 (22.4) | 0.06  0.003  0.01  0.001 |

^#^ p-values are for conversion to MS compared to the first (reference) category or *for any vs. no lesions in that location on MRI

**Supplementary Table 6** Results of the best fitting logistic regression model of the predictors of progression from a first demyelinating event to clinically definite multiple sclerosis in all participants with a first demyelinating event within the study recruitment period, excluding those with data reconstructed from clinical notes.

|  | **All first demyelinating event (n=157)** |  | **First demyelinating event only at baseline interview (n=109)** | |
| --- | --- | --- | --- | --- |
| **Variable** | **aOR (95%CI)** | ***p*** | **aOR (95%CI)** | ***p*** |
| Age at baseline (per 1 year increment) | 0.88 (0.82-0.95) | *0.001* | 0.86 (0.79-0.93) | *<0.001* |
| Current smoker at baseline (yes, vs. no) | 2.97 (0.87-10.21) | *0.08* | 4.75 (1.14-19.75) | *0.03* |
| Leisure time sun exposure from 6-18 years (per 100kJ/m^2^ increment) | 0.87 (0.73-1.03) | *0.11* | 0.87 (0.71-1.06) | *0.17* |
| *HLA-B* (rs2523393)  Any G vs. A:A | 0.12 (0.04-0.43) | *0.001* | 0.12 (0.03-0.52) | *0.005* |
| *TNFRS1A* (rs1800693)  Any C vs. T:T | 8.66 (2.56-29.30) | *0.001* | 12.35 (2.64-57.77) | *0.001* |
| Vitamin D binding protein (rs7041)  Any A vs. C:C | 4.82 (1.55-14.93) | *0.006* | 3.20 (0.88-11.66) | *0.08* |
| Infra-tentorial lesions on MRI (yes. vs. no) | 13.45 (2.75-65.91) | *0.001* | 16.26 (2.66-99.25) | *0.003* |

| Pseudo R^2^, p for the model | 0.42, *p<0.0001* | 0.46, *p<0.0001* |
| --- | --- | --- |

**Supplementary Table 7**. Results of the Cox proportional hazards regression models for individual variables for progression to clinically definite multiple sclerosis (n=196), including for a sensitivity analysis (right hand columns) where those with unknown day or month of clinically definite multiple sclerosis diagnosis or date of first demyelinating event were excluded from the analysis. In the full analysis, unknown day is coded as 15; and unknown month as 6.

|  | **All first demyelinating event** | | | No imputed date data (n=153) | | |
| --- | --- | --- | --- | --- | --- | --- |
|  | AHR* | 95%CI | p | AHR* | 95%CI | p |
| Number of Barkhof criteria | 1.04 | 0.91-1.19 | 0.59 | 1.12 | 0.95-1.32 | 0.20 |
| 25(OH)D (per 10nmol/L) | 1.04 | 0.98-1.10 | 0.17 | 1.06 | 0.99-1.13 | 0.10 |
| Low 25(OH)D (<50nmol/L) | 0.79 | 0.53-1.19 | 0.26 | 0.75 | 0.46-1.23 | 0.26 |
| High 25(OH)D (≥75nmol/L) | 1.14 | 0.83-1.59 | 0.42 | 1.32 | 0.89-1.95 | 0.17 |
| Smoker at baseline(yes/no) | 0.97 | 0.69-1.38 | 0.88 | 1.12 | 0.74-1.69 | 0.59 |
| Total years smoked at baseline | 1.00 | 0.99-1.02 | 0.62 | 1.01 | 0.99-1.02 | 0.49 |
| Human herpes virus 6 immunoglobulin titre category^ | 1.14 | 0.75-1.73 | 0.55 | 1.24 | 0.77-1.99 | 0.38 |
| Epstein Barr virus nuclear antigen immunoglobulin category# | 1.58 | 1.07-2.34 | 0.02 | 1.44 | 0.90-2.32 | 0.13 |
| Leisure ultraviolet radiation dose; 6- current age (per 10^6^ J/m^2^) | 0.70 | 0.55-0.90 | 0.004 | 0.67 | 0.50-0.89 | 0.006 |
| Leisure ultraviolet radiation dose 6-18 years (per 10^5^ J/m^2^) | 0.92 | 0.87-0.97 | 0.003 | 0.89 | 0.83-0.95 | 0.006 |
| Ultraviolet radiation dose 1 month prior to onset of first demyelinating event (per 10^4^ J/m^2^) | 0.61 | 0.25-1.49 | 0.28 | 0.59 | 0.21-1.68 | 0.33 |
| Smoking status  0/0  0/1  1/0  1/1 | 1.00  0.78  2.69  1.17 | 0.24-2.54  1.06-6.83  0.72-1.88 | 0.68  0.04  0.53 | 1.00  0.84  3.04  1.20 | 0.25-2.77  0.89-10.41  0.69-2.10 | 0.77  0.08  0.52 |
| Vitamin D supplement at baseline (yes/no) | 1.28 | 0.85-1.91 | 0.22 | 1.13 | 0.70-1.80 | 0.62 |
| Total T2 lesions at baseline | 1.00 | 0.96-1.05 | 0.87 | 1.02 | 0.97-1.07 | 0.41 |
| Steroid (y/n) at baseline | 1.65 | 1.14-2.40 | 0.009 | 1.55 | 1.01-2.38 | 0.04 |

*Adjusted for age and sex. ^HHV-6 IgG ≤40 vs >40; EBNA IgG ≤160, >160

Smoking status: first number is baseline; 2^nd^ is at follow-up. 0=not smoking; 1=smoking
